# Acetylcholinesterase Biosensor Based On Mesoporous Hollow Carbon Spheres/Core-Shell Magnetic Nanoparticles-Modified Electrode for the Detection of Organophosphorus Pesticides

**DOI:** 10.3390/s18124429

**Published:** 2018-12-14

**Authors:** Ruiping Luo, Zijie Feng, Guannan Shen, Yi Xiu, Yukun Zhou, Xiaodi Niu, Hongsu Wang

**Affiliations:** College of Food Science and Engineering, Jilin University, Changchun 130062, China; luorp16@mails.jlu.edu.cn (R.L.); fengzj9916@mails.jlu.edu.cn (Z.F.); shenguannan@163.com (G.S.); xiuyi17@mails.jlu.edu.cn (Y.X.); zhouyk9916@mails.jlu.edu.cn (Y.Z.)

**Keywords:** mesoporous hollow carbon spheres (MHCS), acetylcholinesterase (AChE), core-shell structure, Malathion

## Abstract

The present study investigated the synthesis of mesoporous hollow carbon spheres (MHCS) and magnetic mesoporous hollow carbon spheres with core-shell structures (Fe_3_O_4_@MHCS). Two acetylcholinesterase sensors (acetylcholinesterase/mesoporous hollow carbon spheres/glassy carbon electrode (AChE/MHCS/GCE) and acetylcholinesterase/core-shell magnetic mesoporous hollow carbon spheres/glassy carbon electrode (AChE/Fe_3_O_4_@MHCS/GCE) based on mesoporous carbon materials were prepared. Under the optimum conditions, using Malathion as the model compound, the developed biosensors showed a wide detection range, low detection limit, good reproducibility, and high stability. The AChE/MHCS/GCE electrochemical sensor response exhibited two good linear ranges at the incubation time of 10 min at the Malathion concentration ranges of 0.01 to 100 ppb and 100 to 600 ppb, with a detection limit of 0.0148 ppb (S/N = 3). The AChE/Fe_3_O_4_@MHCS/GCE electrochemical sensor that was operated with an incubation time of 12 min at the malathion concentration ranges between 0.01–50 ppb and 50–600 ppb had a detection limit of 0.0182 ppb (S/N = 3). Moreover, the AChE/MHCS/GCE and AChE/Fe_3_O_4_@MHCS/GCE biosensors were effective for the detection of real samples, and were demonstrated to be suitable for the field-testing of organophosphorus pesticide (OP) residues.

## 1. Introduction

Organophosphorus pesticides (OPs) and their derivatives are widely used in agricultural applications because of their high efficiencies. However, OPs are potentially toxic for humans and animals as pesticide residues in the environment [1,2]. The primary mechanism behind OP poisoning is that OPs cause changes in the catalytic activity of acetylcholinesterase (AChE) by producing a stable complex at the main part of the AChE [3]. OPs exhibit high toxicity and cause long-term damage to the environment because of the bioaccumulation effect. Therefore, pesticide residue analysis remains an important concern, especially for the detection and quantitative analysis of pesticides [4]. The primary methods that are employed for pesticide analysis include gas chromatography (GC), high-performance liquid chromatography (HPLC), and other spectroscopy methods [5,6]. However, these methods exhibit certain disadvantages, such as long analysis time, cumbersome operation, and high cost [7,8]. Therefore, there is a need for the development of a basic, rapid, and cheap analysis method for the detection of pesticides. In recent years, electrochemical sensors based on AChE have been widely used for the detection of OP residues because of their ideal properties, such as high sensitivity, fast response, low cost, and applicability for field testing [9,10]. The development of such sensors requires suitable materials that could be used as a matrix/transducer for signal conversion and reliable/reproducible detection.

Carbon nanomaterials are suitable candidates for device fabrication because of their high specific surface area and excellent electrocatalytic properties [11]. The degradation of carbon nanomaterials can produce carbon nanotubes, graphene derivatives, and different composites (e.g., nitrogen-doped mesoporous carbon and mesoporous carbon-metal oxides hybrids) [12,13]. Furthermore, the structural design of the material is crucial for enhancing sensor performance. Different structures based on carbon nanomaterials have been successfully synthesized, including nanotubes, nanosheets, nanoboxes, and mesoporous spheres [14,15,16,17,18,19]. Among them, mesoporous spheres were found to exhibit unique characteristics, such as high surface area, high porosity, and good electric conductivity [20]. The magnetic Fe_3_O_4_ nanoparticles possess unique properties such as large surface area, high chemical stability, and low toxicity. Furthermore, functioning as electron-conducting pathways facilitates electron transfer between redox systems and bulk electrode materials [21,22,23]. Moreover, it has been shown in literature studies that Fe_3_O_4_ can maintain stability in various complex backgrounds such as high temperature, high pressure, strong acid, and strong alkali properties, so it has a better application prospect [24,25,26]. Currently, Fe_3_O_4_ nanoparticles have become the component for popular magnetic nanoparticles in many areas of research, such as catalysis, sensing, optoelectronic devices, solar energy cells, and electrode materials [27,28,29,30,31]. Since magnetic Fe_3_O_4_ nanoparticle-based biosensors offer a sensitive tool to quickly detect analytes, several biomolecules such as AChE have been immobilized on iron oxide nanoparticles to construct sensors for the detection of organophosphorus pesticides, presenting excellent performance such as good stability and a lower detection limit [25,32,33]. Recent studies have investigated magnetic nanoparticles that have been coated with various organic or inorganic materials, such as poly (lactic-co-glycolic acid), dextran, and carbon, to improve the stability, hydrophilicity, and biocompatibility [34,35,36]. The coating of carbon materials prevents the agglomeration of magnetic nanoparticles and provides a large area support for further modification. The combination of metal nanoparticles with a large area support, such as carbon spheres, can further improve the properties of the resulting material [37]. In addition, core-shell-structured magnetic mesoporous carbon spheres with magnetic core and porous shells were particularly found to be ideal for various applications because of the highly connected porous structures and well-protected magnetic components [38,39].

**Scheme 1:**
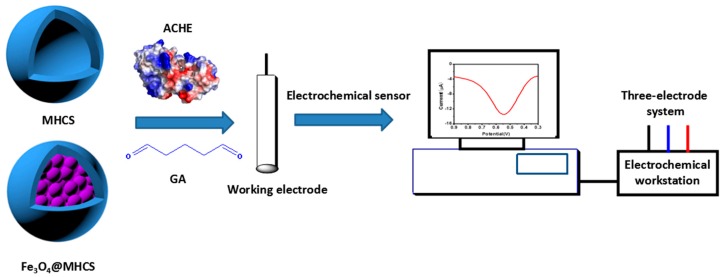
Schematic illustration of the preparation of acetylcholinesterase (AChE)/mesoporous hollow carbon spheres (MHCS)/glassy carbon electrode (GCE) and AChE/ferrosoferric oxide @ mesoporous carbon core-shell structures (Fe_3_O_4_@MHCS)/GCE electrochemical sensors.

In the present study, we synthesized two carbon materials, namely, mesoporous hollow carbon spheres (MHCS) and core-shell magnetic mesoporous hollow carbon spheres (Fe_3_O_4_@MHCS), via the hydrothermal method. Acetylcholinesterase sensors based on the two mesoporous carbon materials were prepared by physical adsorption and glutaraldehyde cross-linking methods. The Fe_3_O_4_ nanoparticles were introduced into mesoporous hollow carbon spheres to construct an acetylcholinesterase sensor in order to achieve the rapid detection of organophosphorus pesticides and compare with the sensor based on hollow carbon sphere materials. This concept of sensors is summarized in Scheme 1. The MHCS and Fe_3_O_4_@MHCS were first synthesized. Next, the working electrodes of the sensors that were modified by the materials (MHCS and Fe_3_O_4_@MHCS), AChE, and glutaraldehyde were further applied for organophosphorus pesticide (Malathion). The properties of the two materials were studied by scanning electron microscopy (SEM), energy dispersive X-ray spectroscopy (EDS), transmission electron microscopy (TEM), X-ray diffraction spectroscopy (XRD), N_2_ adsorption-desorption isotherms, and magnetic hysteresis loops. The parameters affecting the performance, such as mass fraction glutaraldehyde (GA), the pH of the test solution, the carbon:AChE/GA ratio, the mass fraction of the material, and the time of inhibition were optimized. Under optimum conditions, using Malathion as the model compound, the developed biosensors showed a wide range of detection, low detection limit, good reproducibility, and high stability. Moreover, the AChE/MHCS/glassy carbon electrode (GCE) and AChE/Fe_3_O_4_@MHCS/GCE biosensors can be used for the detection of real samples, and were found to be suitable for the field testing of OP residues.

## 2. Materials and Methods

### 2.1. Materials

Iron(III) chloride hexahydrate (FeCl_3_·6H_2_O), dibasic sodium phosphate (Na_2_HPO_4_), ethanol, aqueous ammonia (NH_3_·H_2_O, 25 wt.%), resorcinol, formaldehyde (37 wt.%), hydrofluoric acid (HF, 5 wt.%), tetrapropyl orthosilicate (TPOS), potassium hexacyanoferrate (K_3_[Fe(CN)]_6_), potassium chloride (KCl), glutaraldehyde (GA, 50%), and the above chemical reagents were purchased from the Beijing Chemical Factory reagent company (Beijing, China), and all of the chemicals that were used in this investigation were of analytical grade (99.9%). Potassium phosphate buffer solution (PBS, pH 7.4) was prepared using deionized water (DI water). Acetylcholinesterase (AChE, 500 UN) and acetylthiocholine chloride (ATCl) were purchased from Sigma-Aldrich (St. Louis, MO, USA). The Malathion solution was purchased from Sinopharm Chemical Reagent Co., Ltd. (Shanghai, China). Practical pear samples were purchased from the local market. The AChE and ATCl solution were prepared using PBS and stored at 4 °C for further use. All of the other aqueous solutions were prepared with distilled water.

### 2.2. Synthesis of Mesoporous Carbon Materials

#### 2.2.1. Synthesis of Mesoporous Hollow Carbon Spheres (MHCS)

Mesoporous hollow carbon spheres (MHCS) were synthesized following previously described methods [20]. Briefly, 3.46 mL of TPOS and three mL of NH_3_·H_2_O were slowly and sequentially added to the solution containing ethanol (70 mL) and H_2_O (10 mL), with stirring at room temperature. After 15 min of incubation, 0.4 g of resorcinol and 0.56 mL of formaldehyde were added to the solution, after which the mixture was continuously stirred for 24 h at room temperature. Then, the precipitates were separated by centrifugation at 13,000 rpm·min^−1^, washed with water and ethanol, and dried at 50 °C for 12 h. The materials obtained above were subjected to carbonization at 700 °C (2 °C·min^−1^) under N_2_ atmosphere for five hours. After the removal of silica by hydrofluoric acid (HF), the final MHCS products were obtained.

#### 2.2.2. Synthesis of Ferrosoferric Oxide @ Mesoporous Carbon Core-Shell Structures (Fe_3_O_4_@MHCS)

First, Fe_2_O_3_ was prepared according to a previously described method [40]. FeCl_3_·6H_2_O (2 mmol) and sodium phosphate (NaH_2_PO_4_, 0.02 mmol) were dissolved in 100 mL of EtOH/H_2_O solution (1:1 in volume) with stirring. Afterwards, the mixture was subjected to hydrothermal treatment at 100 °C for 48 h. The solid products were collected by centrifugation, washed with distilled water and ethanol, and subsequently dried at 50 °C overnight. The Fe_2_O_3_ was obtained. Then, 30 mg of Fe_2_O_3_ was dispersed in a solution containing ethanol (70 mL), distilled water (10 mL), and ammonia water (three mL, 25 wt.%) by sonication. Next, TPOS (0.5 mL), resorcinol (0.1 g), and formaldehyde (0.14 mL, 37 wt.%) were added to the solution, and the resulting mixture was stirred continuously for 24 h. The precipitates were separated by centrifugation, washed with water and ethanol, and dried at 50 °C overnight. Fe_3_O_4_@MHCS yolk-shell structures were obtained after carbonization at 700 °C under N_2_ for 5 h and the removal of silica by NaOH solution (4 M) [20].

### 2.3. Characterization

The morphology, compositions, and structure of the above-synthesized materials were investigated by a JEOL Hitachi S-4800 scanning electron microscopy (SEM) (Hitachi S-4800, Tokyo, Japan) with an accelerating voltage of 10 KV, and transmission electron microscopy (TEM, Tecnai G2F30, FEI, Hillsboro, OR, USA) with operating voltage at 100 KV, equipped with an energy dispersive spectral (EDS) analyzer (Hitachi, Tokyo, Japan). X-ray diffraction (XRD) analysis was conducted using a Bruker High-Resolution D8 Advance XRD unit (Bruker, Karlsruhe, Germany). N_2_ adsorption-desorption isotherms were measured using a Micromeritics ASAP 2020 analyzer (Micromeritics, Atlanta, GA, USA). The surface functional groups of the carbon materials were measured by Fourier transform infrared (FTIR) spectroscopy (IR Prestige-21, Shimadzu, kyoto, Japan). The magnetic hysteresis loop was measured using an MPMS (Quantum Design, San Diego City, CA, USA) at temperatures of 300 K.

The electrochemical experiments were performed using a Chenhua CHI660E electrochemical workstation (Shanghai Chenhua Instrument Co., Ltd., Shangha, China). All of the electrochemical studies were performed with a conventional three-electrode system. A glassy carbon electrode (GCE), an Ag/AgCl (3 M KCl) electrode, and a Pt foil electrode were used as the working electrode, reference electrode, and the counter electrode, respectively. Typical cyclic voltammograms (CV) were obtained in 5.0 mM of [Fe(CN)_6_]^3−^ containing 0.1 M of KCl. The scanning range was from 0.6 V to −0.1 V, and the scanning rate was 100 mV·s^−1^. The electrochemical impedance spectroscopy (EIS) measurements were performed in five mM of [Fe(CN)_6_]^3−^ containing 0.1 M of KCl. The amplitude of the applied sine wave potential was 3.2 mV. The impedance measurements were recorded at a bias potential of 180 mV within the frequency range of 0.01 to 10 kHz. The DPV measurements were conducted in different concentrations Malathion solution, with the scanning range from 0.3 to 0.8 V.

### 2.4. Fabrication of the Working Electrode

Before fabrication, the GCE was polished until obtaining a mirror-like surface using 0.3-μm and 0.05-μm alumina slurries, followed by a thorough rinsing with ethanol and deionized water. For preparing the modified electrode, 6 μL of mixed solution containing carbon materials (MHCS and Fe_3_O_4_@MHCS, respectively), AChE/GA (2:1) was overcoated on GCE by drop casting and allowed to dry at 4 °C. The modified electrodes were designated as AChE/MHCS/GCE and AChE/Fe_3_O_4_@MHCS/GCE, and were used for subsequent studies.

### 2.5. Measurement of Inhibition

The AChE/MHCS/GCE and AChE/Fe_3_O_4_@MHCS/GCE electrodes were incubated in PBS solution (0.1 M) containing different concentrations of Malathion for a few minutes, after which the biosensor surface was rinsed with PBS. The peak currents of the original (*I*_0_) and inhibitory (*I*_1_) electrodes in PBS containing acetylthiocholine chloride (ATCl) were recorded. The inhibition of the pesticide Malathion was calculated as follows:

*I*_0_ = [(*I*_0_ − *I*_1_)/*I*_0_] × 100%

### 2.6. Preparation and Determination of Real Samples

Fruit samples (pears) were obtained from a local supermarket and cut after washing three times with double-distilled water. Following the procedure previously described by Yu et al. [41]. 10 g of samples were placed in a beaker, to which 50 mL of acetone/0.1 M PBS solution (volume ratio of 1:9) was added; then, it was stirred for 30 min, and subsequently centrifuged to obtain the supernatant. According to the spike method, 10, 50, and 200 ppb of Malathion solution was added dropwise to the supernatant. The resulting product was used for electrochemical detection and determination of the oxidation peak current.

### 2.7. Precision and Stability of the Biosensors

The precision between the electrodes and within the electrode was demonstrated by measuring the peak current values of the six electrodes and one electrode six times at the same concentration of Malathion solution, respectively. The stability was demonstrated by measuring the peak current values of the AChE/MHCS/GCE and AChE/Fe_3_O_4_@MHCS/GCE on the first day and stored at 4 °C for 30 days.

## 3. Results and Discussion

### 3.1. Characterization of Materials

**Figure 1:**
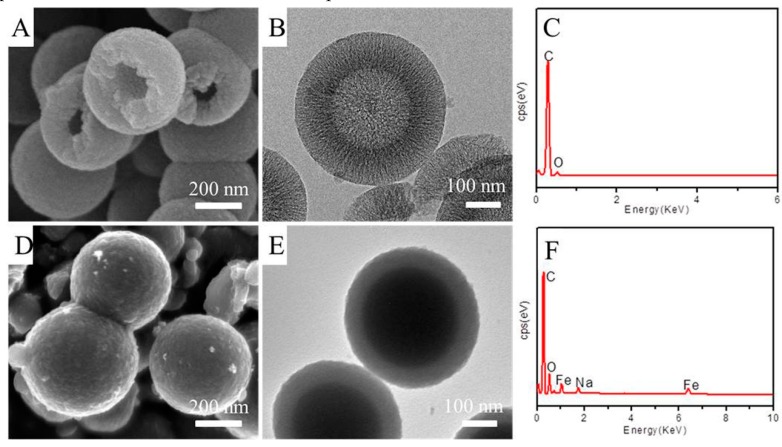
(**A**) Scanning electron microscopy (SEM) of the MHCS; (**B**) Transmission electron microscopy (TEM) of the MHCS; (**C**) Energy dispersive X-ray spectroscopy (EDS) of the MHCS; (**D**) SEM of the Fe_3_O_4_@MHCS; (**E**) TEM of the Fe_3_O_4_@MHCS; (**F**) EDS of the Fe_3_O_4_@MHCS.

Figure 1 showed representative SEM images and TEM images of the morphology of the as-prepared MHCS and Fe_3_O_4_@MHCS. In the SEM image, the MHCS were observed as perfect spheres with hollow morphologies (Figure 1A). In addition, the Fe_3_O_4_@MHCS exhibited good spherical structures, and some substances in the cavity can be observed (Figure 1D). From Figure 1C,F, the EDS revealed that the MHCS sample mainly contained two elements (C, O), and the Fe_3_O_4_@MHCS sample mainly contained three elements (C, Fe, and O), indicating that Fe_3_O_4_ was successfully introduced to MHCS. The MHCS and Fe_3_O_4_@MHCS particles had a uniform diameter of about 400 nm and a radial porous shell with a thickness of ~100 nm. The hollow structures and the radial pore channels were evident in the TEM image (Figure 1B,E). The Fe_3_O_4_@MHCS evidently showed a core-shell structure, which further demonstrated the successful synthesis of the Fe_3_O_4_@MHCS particles (Figure 1E). Considering the mesoporous structures on the surface of the sphere, the immobilization of AChE could be realized.

**Figure 2:**
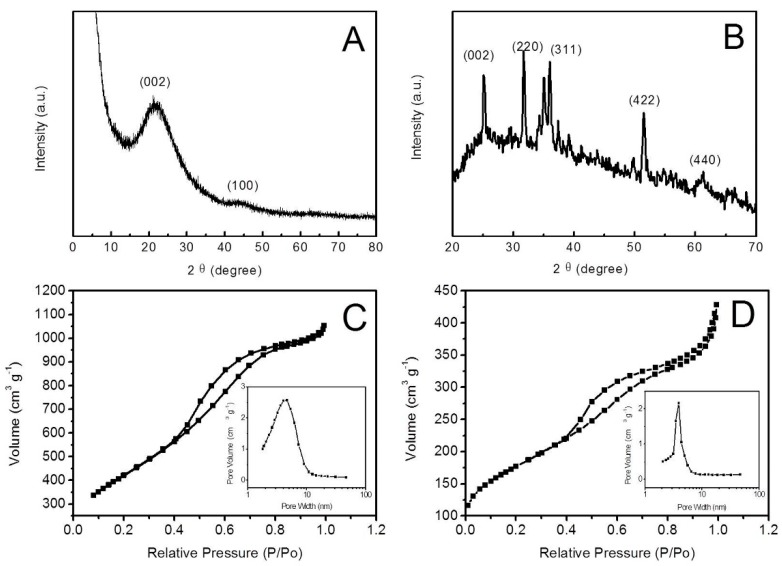
X-ray diffraction (XRD) patterns of MHCS (**A**) and Fe_3_O_4_@MHCS (**B**); Nitrogen adsorption-desorption isotherm of MHCS (**C**) and Fe_3_O_4_@MHCS (**D**). Inset: pore size distribution curve.

The XRD patterns and N_2_ sorption isotherms of MHCS and Fe_3_O_4_@MHCS were shown in Figure 2 to verify the structure of the materials. The two characteristic diffraction peaks of C were present in the MHCS samples (Figure 2A). The weak diffraction peaks of MHCSs that were observed at 22.3° (2θ) and 42.5° (2θ) were indexed as the crystal planes (002) and (100), which were assigned to graphitic carbon, further demonstrating the successful synthesis of the MHCS [42]. As shown in Figure 2B, the diffraction peaks of Fe_3_O_4_@MHCS at 25.2°, 31.7°, 35.9°, 53.4°, and 62.5° correspond to the diffraction peaks (002), (220), (311), (422), and (440). The (002) diffraction peak of carbon appeared at 22.3° in the MHCSs, indicating that Fe_3_O_4_ doping caused the peak to shift to the right. The diffraction peaks (220), (311), (422), and (440) correspond to the typical Fe_3_O_4_ spinel (JCPDS01-1111), indicating that the Fe_3_O_4_ can be well preserved during the carbonization process [43]. As shown in Figure 2C,D, both MHCS and Fe_3_O_4_@MHCS showed typical type IV curves with an H1 hysteresis loop that is typical of mesoporous materials. The adsorption capacity of the MHCSs was considerably higher than that of the Fe_3_O_4_@MHCS, which is primarily explained by the doping of Fe_3_O_4_ that blocks the cavity of the MHCS. In addition, the pore size distributions of the MHCS and Fe_3_O_4_@MHCS particles clearly suggested that the distribution of the MHCS was centered at 4.10 nm, whereas the distribution of Fe_3_O_4_@MHCS was centered at 3.90 nm. Additionally, MHCS possessed a high surface area of 1525.40 m^2^·g^−1^ and a total pore volume of 1.57 cm^3^·g^−1^, which were markedly higher than the surface area of 601.20 m^2^·g^−1^ and pore volume of 0.64 cm^3^·g^−1^ of Fe_3_O_4_@MHCS. The surface area, pore volume, and pore size of Fe_3_O_4_@MHCS particles were lower than those of MHCS particles, indicating that Fe_3_O_4_ was successfully doped.

**Figure 3:**
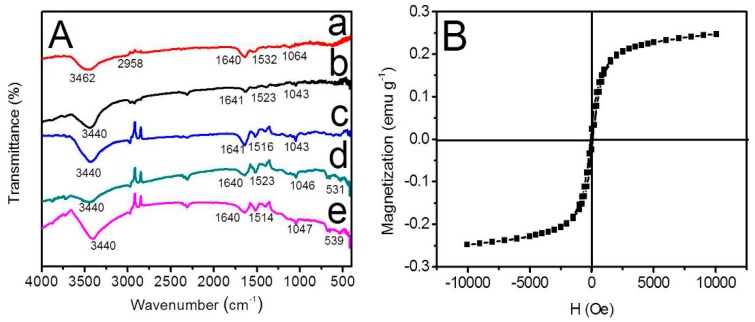
(**A**) Fourier transform infrared (FTIR) spectra of AChE (a), MHCS (b), AChE/MHCS (c), Fe_3_O_4_@MHCS (d), and AChE/Fe_3_O_4_@MHCS (e); (**B**) the hysteresis loops of Fe_3_O_4_@MHCS.

The functional groups located on the surface of AChE, MHCS, AChE/MHCS, Fe_3_O_4_@MHCS, and AChE/Fe_3_O_4_@MHCS were identified by Fourier transform infrared (FTIR) spectroscopy (Figure 3A). The AChE (a) that was observed had –OH absorption peaks at 3420 cm^−1^ and 1040 cm^−1^, amide bond absorption peaks at 1640 cm^−1^ and 1532 cm^−1^, and a C–H absorption peak at 2958 cm^−1^. It can be seen that MHCS (b) had bands observed at 3440 cm^−1^, 1641 cm^−1^, 1523 cm^−1^, and 1043 cm^−1^, corresponding to the absorption peak of O–H, C=C, C–H, C–O and O–H, respectively. Figure 3A plot c showed the FTIR spectra of MHCS after immobilization of the AChE, from which can be observed that it had the same groups as the MHCS particle. However, the hydroxyl peak and the amide peak area increased, indicating that the AChE enzyme was successfully immobilized. The Fe_3_O_4_@MHCS samples (d) had bands at 3440 cm^−1^, 1640 cm^−1^, 1523 cm^−1^, 1050 cm^−1^, and 531 cm^−1^, which correspond to the stretching vibration absorption peaks of O–H, C=C, C–H, C–O, O–H, and Fe–O, respectively. The appearance of Fe–O characteristic vibration peaks proved that Fe_3_O_4_ was successfully introduced to MHCS, and Fe_3_O_4_@MHCS was successfully synthesized [44]. Similarly, the AChE/Fe_3_O_4_@MHCS (e) had the same bands as Fe_3_O_4_@MHCS (d), and the hydroxyl peak area increased, indicating that the AChE enzyme was successfully immobilized to Fe_3_O_4_@MHCS samples. The field-dependent magnetization curve of the Fe_3_O_4_@MHCS nanoparticles was investigated using a super conducting quantum interference device (SQUID) magnetometer operated at 300 K. Results revealed that the nanocomposites have a saturation magnetization of 0.25 emu·g^−1^, which indicated the presence of an effective ferromagnetic state (Figure 3B).

### 3.2. Electrochemical Behavior of the AChE Biosensor

**Figure 4:**
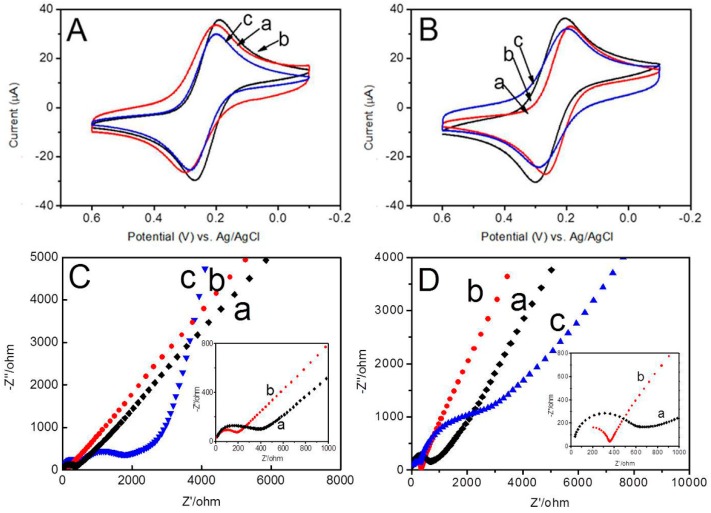
(**A**) Cyclic voltammogram (CV) curves of the GCE (a), MHCS/GCE (b), AChE/MHCS/GCE (c); (**B**) CV curves of the GCE (a), Fe_3_O_4_@MHCS/GCE (b), AChE/Fe_3_O_4_@MHCS/GCE (c); (**C**) electrochemical impedance spectroscopy (EIS) of the GCE (a), MHCS/GCE electrode (b), AChE/MHCS/GCE (c); (**D**) AC impedance spectra of the GCE (a), Fe_3_O_4_@MHCS/GCE (b), AChE/Fe_3_O_4_@MHCS/GCE (c). All tests were conducted in 5.0 mM [Fe(CN)_6_]^3−^ containing 0.10 M of KCl: Scanning speed: 100 mV·s^−1^.

Cyclic voltammetry (CV) and electrochemical impedance spectroscopy (EIS) are commonly employed methods for electrochemical characterization. Figure 4 showed the CV curves and EIS spectra of the two developed sensors, which were detected in a 5.0 mM [Fe(CN)_6_]^3−^ (containing 0.10 M KCl) solution. The CV curves of the GCE (a), MHCS/GCE (b), and the AChE/MHCS/GCE (c) were presented in Figure 4A. Curve (a) shows that the GCE had a pair of redox peaks with good reversibility. Compared with the GCE, the redox peak of curve (b) was significantly higher, which was mainly attributed to the bare electrode modified by MHCS particles to enhance the electron transfer on the electrode surface. The redox peak of curve (c) was significantly lower compared to that of the MHCS/GCE electrode, which could be explained by the non-conductivity of AChE, which prevented electron transfer on the electrode surface. These results indicated that the AChE was successfully fixed on the electrode surface. Similarly, the impedance value of the modified electrode MHCS/GCE (b) was 160 Ω, which was considerably smaller than that of the GCE at 300 Ω (a) in Figure 4C. The impedance value increased to 2500 Ω (c) after the AChE was fixed, which also proved that AChE was successfully immobilized on the electrode surface.

As shown in Figure 4B, curves (a), (b), and (c) represented GCE, Fe_3_O_4_@MHCS/GCE, and AChE/Fe_3_O_4_@MHCS/GCE, respectively. These curves followed a similar trend as that observed with the AChE/MHCS/GCE sensors, thereby demonstrating that Fe_3_O_4_@MHCS can enhance the transmission of electrons and increase the conductivity of the electrode. Therefore, the fixation of AChE in the Fe_3_O_4_@MHCS/GCE reduced the conductivity. As shown in Figure 4D, the impedance values of the GCE (a), Fe_3_O_4_@MHCS/GCE (b), and AChE/Fe_3_O_4_@MHCS/GCE (c) were 600, 200, and 3300 Ω, respectively. The above findings further demonstrated that the Fe_3_O_4_@MHCS particles could effectively increase the conductivity of the electrode and the AChE.

**Figure 5:**
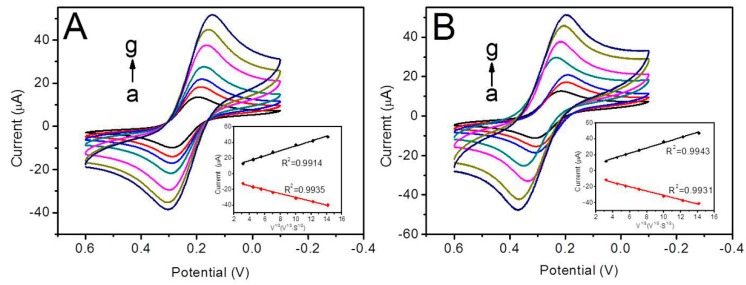
CVs of AChE/MHCS/GCE (**A**) and AChE/Fe_3_O_4_@MHCS/GCE (**B**) in 5.0 mM of [Fe(CN)_6_]^3−^ containing 0.10 M KCl obtained at different scan rates of 10 mV·s^−1^ (a), 20 mV·s^−1^ (b), 30 mV·s^−1^ (c), 50 mV·s^−1^ (d), 100 mV·s^−1^ (e), 150 mV·s^−1^ (f), and 200 mV·s^−1^ (g). The inset shows the linear relationship between the current and the square root of the scanning speed.

The CV curves of the AChE/MHCS/GCE and AChE/Fe_3_O_4_@MHCS/GCE biosensors in 5.0 mM [Fe(CN)_6_]^3−^ containing 0.10 M of KCl solution at different scan rates were presented in Figure 5. A linear relationship was observed between the peak current and the square root of the different sweep speeds at 10–200 mV·s^−1^. Increasing the scan rate led to a shift in the oxidation peak potential; the peak current increased linearly with the square root of the scan rate, indicating that the electrochemical behavior of the sensor was controlled by diffusion [45].

**Figure 6:**
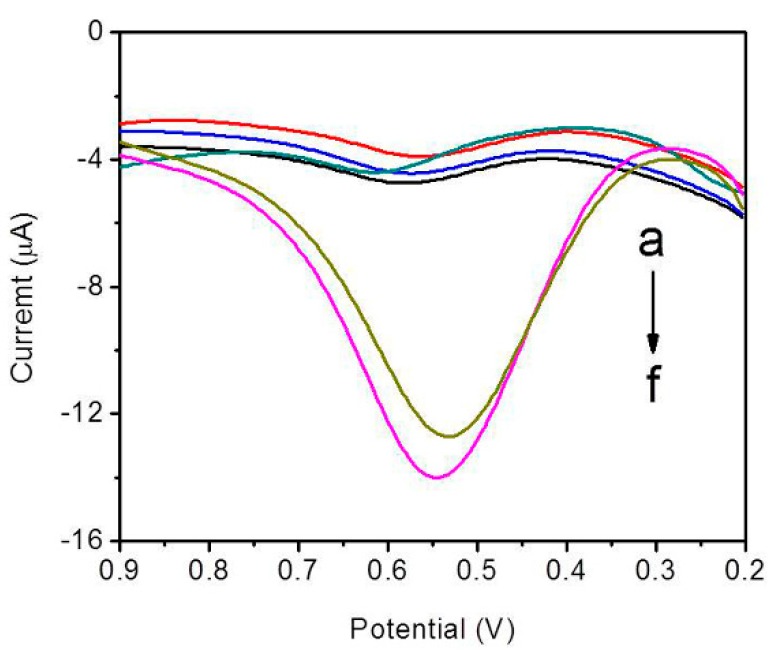
Differential pulse voltammetry (DPV) of AChE/MHCS/GCE in 0.1 M PBS solution (no ATCl) (a); AChE/Fe_3_O_4_@MHCS/GCE in 0.1 M of phosphate buffer solution (PBS) (no ATCl) (b), MHCS/GCE in 1.5 mM of ATCl solution (c), Fe_3_O_4_@MHCS/GCE in 1.5 mM of ATCl solution (d), AChE/Fe_3_O_4_@MHCS/GCE in 1.5 mM of ATCl solution (e) and AChE/MHCS/GCE (f) in 1.5 mM of ATCl solution.

Figure 6 showed the differential pulse voltammetry (DPV) behaviors of different trim electrodes. In the PBS (0.01 M, pH 7.2) solution, the AChE/MHCS/GCE and AChE/Fe_3_O_4_@MHCS/GCE sensors had no reaction peaks (a, b). Similarly, the MHCS/GCE (c) and Fe_3_O_4_@MHCS/GCE (d) had no peaks in the PBS solution containing 1.5 mM of ATCl, indicating that the two materials prepared and ATCl did not undergo a catalytic reaction. The AChE/Fe_3_O_4_@MHCS/GCE (e) and the AChE/MHCS/GCE (f) produced significant peaks in the PBS solution containing ATCl, indicating the occurrence of a catalytic reaction in the electrolytic cell. This peak was produced by AChE, which catalyzed the oxidation of the hydrazinoline of ATCl.

### 3.3. Optimization Parameters of the Biosensor Performance

**Figure 7:**
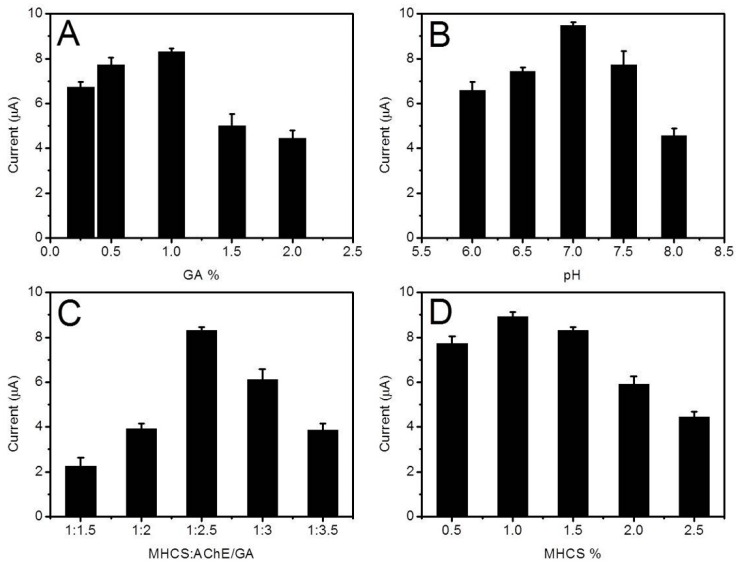
The column chart of the peak current value with concentration of GA (**A**), pH (**B**), and volume ratio of the MHCS:AChE/GA (**C**) and concentration of MHCS (**D**) in 0.1 M of PBS solutions containing 1.6 mM of ATCl.

**Figure 8:**
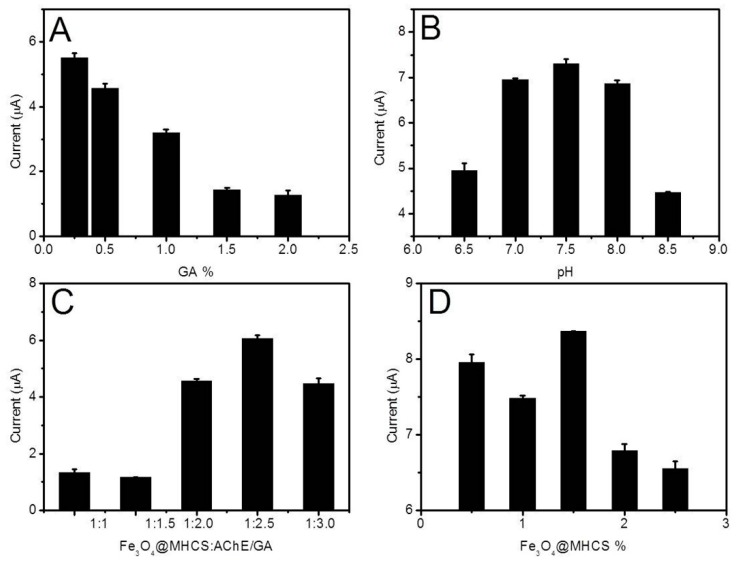
The column chart of the peak current value with concentrations of GA (**A**), pH (**B**), and volume ratio of the Fe_3_O_4_@MHCS:AChE/GA (**C**) and concentration of Fe_3_O_4_@MHCS (**D**) in 0.1 M of PBS solutions containing 1.2 mM of ATCl.

To determine the optimal conditions for the preparation of the sensors, various factors were investigated, including glutaraldehyde (GA) concentration, pH, ratio of carbon:AChE/GA, and material mass fraction. GA was found to be a highly effective cross linker; however, it could destroy the active site of the enzyme [46]. The optimal concentrations of GA in the AChE/MHCS/GCE and AChE/Fe_3_O_4_@MHCS/GCE sensors were 1.0% and 0.25%, respectively (Figure 7A and Figure 8A). However, the enzyme was susceptible to falling off from the surface of the AChE/Fe_3_O_4_@MHCS/GCE sensors at 0.25% GA concentration; therefore, we selected the GA concentration of 0.5% in subsequent experiments. The best mass fraction of GA in AChE/Fe_3_O_4_@MHCS/GCE was lower, and could be attributed to the magnetic properties of Fe_3_O_4_@MHCS, which exerted an adsorption effect on the immobilized AChE. Furthermore, as shown in Figure 7A and Figure 8A, the response current value decreased when the GA concentration exceeded the optimum concentration, which could be attributed to the denaturation of the immobilized enzyme caused by the high GA concentration.

The pH of the substrate solution (ATCl) markedly influenced the enzyme activity; therefore, the AChE/MHCS/GCE and AChE/Fe_3_O_4_@MHCS/GCE electrochemical sensors were operated at different pH values of 6.5, 7.0, 7.5, 8.0, and 8.5. As shown in Figure 7B, the peak current value of the AChE/MHCS/GCE electrochemical sensor in a solution containing 1.6 mM of ATCl reached a maximum current of 9.3 μA at pH 7.0; in addition, the peak current increased at pH values above 7.0. Therefore, the optimum pH of the AChE/MHCS/GCE electrochemical sensor substrate solution was determined to be 7.0. As shown in Figure 8B, the AChE/Fe_3_O_4_@MHCS/GCE electrochemical sensor has a maximum peak current of 7.4 μA in a solution containing 1.2 mM of ATCl at pH 7.5, indicating that the optimum pH of the AChE/Fe_3_O_4_@MHCS/GCE sensor substrate solution was 7.5.

The immobilization of the enzyme affects the peak current value of the electrochemical sensor. Therefore, we examined the varying carbon:AChE/GA ratios of 1:1.5, 1:2, 1:2.5, 1:3, and 1:3.5 on the working electrode to study. The AChE/MHCS/GCE and AChE/Fe_3_O_4_@MHCS/GCE sensors were operated in ATCl solutions containing 1.6 mM of ATCl at pH 7.5 and 1.2 mM of ATCl at pH 7.0. As shown in Figure 7C, at the GA/AChE ratio of 1:2.5, the peak current reached the maximum value, and the response signal decreased when the amount of enzyme was increased, which could be attributed to the increased thickness of the enzyme film. Therefore, in the AChE/MHCS/GCE sensor, the MHCS:AChE/GA ratio of 1:2:5 was selected to achieve the highest sensitivity in subsequent experiments. Similarly, as shown in Figure 8C, the response current value reached the maximum value when the GA/AChE ratio was 1:2.5 in the AChE/Fe_3_O_4_@MHCS/GCE electrochemical sensor.

Figure 7D and Figure 8D showed the effect of the loading amount of MHCS and Fe_3_O_4_@MHCS on the amperometric response of the biosensors, respectively. Initially, increasing the MHCS significantly improved the response current, indicating that the addition of more MHCS improved the sensor performance; furthermore, the response current reached the maximum value at 1.0% MHCS. Further increasing the loading amount of MHCS resulted in a decrease of the response current, which could be attributed to the higher resistance and double-layer capacitance of the modified electrode. Similarly, as shown in Figure 8D, the response current value reached the maximum when the Fe_3_O_4_@MHCS was 1.5% in the AChE/Fe_3_O_4_@MHCS/GCE sensor.

**Figure 9:**
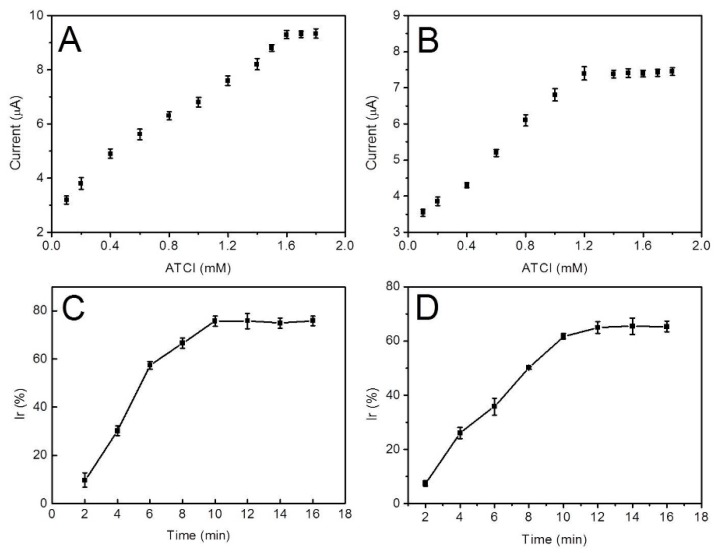
Relationship between the peak response current and concentration of ATCl of AChE/MHCS/GCE (**A**), AChE/Fe_3_O_4_@MHCS/GCE (**B**); Effect of incubation time on the response of ATCl after the AChE/MHCS/GCE (**C**) and AChE/Fe_3_O_4_@MHCS/GCE (**D**) was incubated with 50 ppb of malathion.

In addition, the ATCl concentration was found to affect the sensitivity and stability of the acetylcholinesterase sensor. To determine the optimal concentration of the ATCl substrate, two electrochemical sensors were tested at ATCl concentrations ranging from 0.1 mM to 1.8 mM in 0.1 M of PBS solution. As shown in Figure 9A,B, the peak current increased gradually with increasing ATCl concentration, and reached the maximum value at the ATCl concentrations of 1.6 mM and 1.2 mM. Therefore, 1.6 and 1.2 mM of ATCl were selected as the optimum substrate concentrations for the AChE/MHCS/GCE and AChE/Fe_3_O_4_@MHCS/GCE sensor, respectively.

To determine the relationship between the pesticide inhibition rate and pesticide incubation time, the AChE/MHCS/GCE and AChE/Fe_3_O_4_@MHCS/GCE electrochemical sensors were immersed in a 50 ppb Malathion solution, and the inhibition rate of the pesticide on the acetylcholinesterase sensor was determined. The effects of inhibition time on the sensors were presented in Figure 9C,D. The inhibition rate gradually increased with the prolonged inhibition time until it reached equilibrium, indicating that the binding site between the dethiophos and AChE is balanced. The equilibrium times for the AChE/MHCS/GCE and AChE/Fe_3_O_4_@MHCS/GCE electrochemical sensors were 10 and 12 min, respectively.

### 3.4. Detection of Pesticides

**Figure 10:**
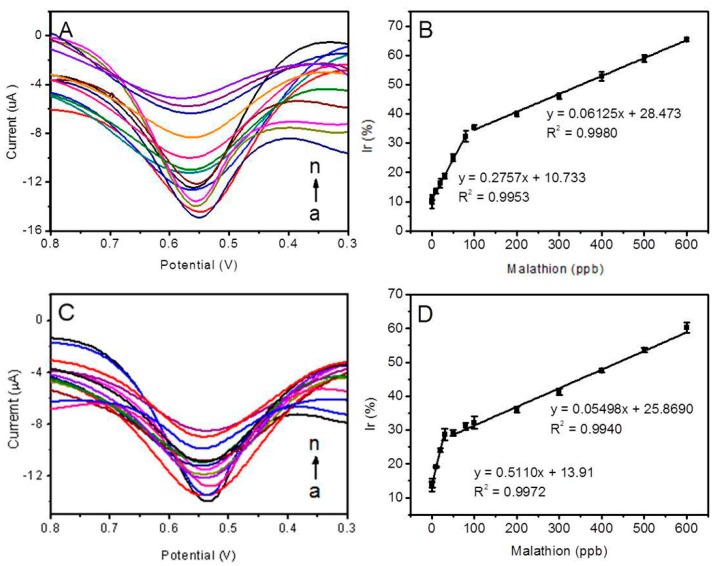
(**A**) DPV of AChE/MHCS/GCE at pH 7.5 in PBS containing 1.6 mM of ATCl after immersion in a malathion solution with different concentrations of 0.01 ppb (a), 0.1 ppb (b), 1 ppb (c), 10 ppb (d), 20 ppb (e), 30 ppb (f), 50 ppb (g), 80 ppb (h), 100 ppb (i), 200 ppb (j), 300 ppb (k), 400 ppb (l), 500 ppb (m), and 600 ppb (n); (**B**) Linear relationships between the inhibition rate and malathion concentration in the AChE/MHCS/GCE sensors; (**C**) DPV of AChE/Fe_3_O_4_@MHCS/GCE at pH 7.0 in PBS containing 1.2 mM of ATCl after immersion in a malathion solution with different concentrations of 0.01 ppb (a), 0.1 ppb (b), 1 ppb (c), 10 ppb (d), 20 ppb (e), 30 ppb (f), 50 ppb (g), 80 ppb (h), 100 ppb (i), 200 ppb (j), 300 ppb (k), 400 ppb (l), 500 ppb (m), and 600 ppb (n); (**D**) Linear relationships between the inhibition rate and malathion concentration in the AChE/Fe_3_O_4_@MHCS/GCE sensors.

We investigated the relationship between the AChE/MHCS/GCE and AChE/Fe_3_O_4_@MHCS/GCE electrochemical sensors and the pesticide concentrations under optimal experimental conditions. The DPV responses were examined before and after exposure to varying concentrations of pesticides. The DPV diagrams of malathion were shown in Figure 10A,C. As the concentration of pesticides increased gradually, the current gradually decreased (curves (a)–(n)). The relationships between the inhibition rate and malathion concentrations are shown in Figure 10B,D, respectively. At the pesticide concentrations ranging from 0.01 to 100 ppb, the inhibition rate of the AChE/MHCS/GCE sensor was found to be linear with the pesticide concentration. The corresponding linear equation was determined to be *y* = 0.2757*x* + 10.73 (*R*^2^ = 0.9953). At pesticide concentrations ranging from 100 to 600 ppb, the inhibition rate was found to be linear with the pesticide concentration. The corresponding linear equation was determined to be *y* = 0.06125*x* + 28.47 (*R*^2^ = 0.9980). The detection limit of the AChE/MHCS/GCE sensor for malathion pesticides was calculated to be 0.0148 ppb (S/N = 3). As shown in Figure 10D, the inhibition rate of the AChE/Fe_3_O_4_@MHCS/GCE sensor was found to be linear with the pesticide concentration. When the concentration of pesticide ranged from 0.01 to 50 ppb, the linear equation was determined to be *y* = 0.5110*x* + 13.91 (*R*^2^ = 0.9972); at the pesticide concentrations ranging from 50 to 600 ppb, the linear equation was determined to be *y* = 0.05498*x* + 25.86 (*R*^2^ = 0.9940). The detection limit of the AChE/Fe_3_O_4_@MHCS/GCE electrochemical sensor for malathion was calculated to be 0.0182 ppb (S/N = 3), which was considerably lower than previously reported values (Table 1). Compared with the two sensors, the AChE/MHCS/GCE sensor had a lower detection limit, which may be due to the unique hollow structure of MHCS. Hollow cores may be more beneficial to the action of enzymes and substrates.

### 3.5. Precision and Stability of the Biosensors

The interassay precision of the AChE/MHCS/GCE electrochemical sensor was estimated by determining the responses of six different electrodes at [ATCl] = 1.6 mM. Six working electrodes were immersed in 50 ppb of malathion for 10 min, and detection was performed with 1.6 mM of ATCl at pH 7.5. The interassay relative standard deviation (RSD) was determined to be 5.6%. Additionally, one of the electrodes was tested six times with the corresponding RSD value of 7.1%, which demonstrated that the AChE/MHCS/GCE sensor had good precision. During the first week of storage, we observed no obvious decrease in performance, and the sensor retained 76% of its initial current response after a 30-day storage period, thereby demonstrating that the AChE/MHCS/GCE sensor exhibited good stability. Similarly, the same method was used to determine the precision and stability of the AChE/Fe_3_O_4_@MHCS/GCE sensor. The relative standard deviations between the electrodes were determined to be 6.4% and 6.9%, respectively. During the first week of storage, we observed no obvious decrease in performance, and the sensor retained 79% of its initial current response after a 30-day storage period. Therefore, similar to the AChE/MHCS/GCE sensor, the AChE/Fe_3_O_4_@MHCS/GCE sensor had excellent precision and stability. The sensor with Fe_3_O_4_ had higher stability than the AChE/MHCS/GCE sensor, which is consistent with reports in the literature [2,37]. The introduction of Fe_3_O_4_, which was capable of maintaining high stability under complex conditions, may be a major cause of long-term stability.

### 3.6. Determination of Real Samples

To further demonstrate the practical applicability of the AChE/MHCS/GCE and AChE/Fe_3_O_4_@MHCS/GCE sensors, recovery tests were performed using pear samples containing 10, 50, and 200 ppb of malathion. The results are summarized in Table 2. The percent recoveries ranged from 97.80% to 104.10%, which indicated that the proposed biosensors were highly accurate, and could be used for the direct analysis of real samples. Furthermore, the electrochemical detection results were consistent with the results of HPLC analysis.

## 4. Conclusions

In summary, we successfully synthesized MHCS and magnetic Fe_3_O_4_@MHCS particles. The properties of the two materials were studied by SEM, TEM, XRD, N_2_ adsorption-desorption analyses, and magnetic hysteresis loop. The AChE/MHCS/GCE and AChE/Fe_3_O_4_@MHCS/GCE sensors, which were developed based on two mesoporous carbon materials, were successfully prepared. The parameters affecting the performance, such as the mass fraction GA, pH of the ATCl, carbon:AChE/GA ratio, mass fraction of the material, and the inhibition period, were optimized. The optimal conditions for the operation of the AChE/MHCS/GCE and AChE/Fe_3_O_4_@MHCS/GCE biosensors were GA concentrations of 1.0% and 0.25%, pH values of 7.0 and 7.5, a carbon:AChE/GA ratio of 1:2.5, and carbon contents of 1.0% and 1.5%. Under these optimum conditions, using malathion as the model compound, the two biosensors exhibited a low detection limit, wide linear range, and good stability. In addition, the AChE/MHCS/GCE electrochemical sensor response showed two good linear ranges during an incubation time of 10 min at the malathion concentrations ranging between 0.01–100 ppb and 100–600 ppb, with a detection limit of 0.0148 ppb (S/N = 3). The AChE/Fe_3_O_4_@MHCS/GCE electrochemical sensor operated at the incubation time of 12 min showed good detection at the malathion concentration ranges between 0.01–50 ppb and 50–600 ppb, with a detection limit of 0.0182 ppb (S/N = 3). Furthermore, the prepared sensors showed good stability, especially with the introduction of magnetic Fe_3_O_4_ nanoparticles, which increased the sensor stability. Moreover, AChE/MHCS/GCE and AChE/Fe_3_O_4_@MHCS/GCE biosensors could be used for the effective detection of real samples, and were found to be suitable for field-testing OP pesticide residues. In particular, the AChE/Fe_3_O_4_@MHCS/GCE electrochemical sensor can be applied to a wider range of actual samples due to its good stability.

## Figures and Tables

**Table 1:** Comparison of the analytical methods for the detection of organophosphorus pesticides (OPs).

Electrode	Pesticide	Linear Range	Detection Limit (ppb)	Reference
AChE/prussian blue (PB) sensor	aldicarb	-	30	[47]
	paraoxon		10	
	parathion-methyl		5	
nafion-silver decorated amine functionalized reduced graphene oxide (NA/rGO-NH_2_)/AChE/GCE	malathion	0.0063–0.077 ppm	4.5	[48]
	methidathion	0.012–0.105 ppm	9.5	
	chlorpyrifos ethyl	0.02–0.122 ppm	14	
chitosan solution (CS)@TiO_2_-CS/reduced graphene oxide (rGO)/GCE	dichlorvos	7.9–4959 ppb	6.4	[49]
AChE/3-carboxyphenylboronic (CPBA)/gold nanoparticles (AuNPs)/reduced graphene oxide-chitosan solution (RGO-CS)/GCE	Isoprocarb	2–10 ppb 20–150 ppb	0.5	[50]
AChE/MHCS/GCE	malathion	0.01–100 ppb 100–600 ppb	0.0148	This work
AChE/Fe_3_O_4_@MHCS/GCE	malathion	0.01–50 ppb 50–600 ppb	0.0182	This work

**Table 2:** Recoveries of malathion in pear samples by AChE/MHCS/GCE and AChE/Fe_3_O_4_@MHCS/GCE sensors.

Sensors	Samples	Added (ppb)	Found (ppb)	Recovery (%)	RSD (%, *n* = 6)
AChE/MHCS/GCE	pear	10	10.41	104.10	3.21
		50	50.76	100.75	4.25
		200	198.45	99.23	3.28
AChE/Fe_3_O_4_@MHCS/GCE	pear	10	9.78	97.80	2.85
		50	51.41	102.82	1.88
		200	201.92	100.96	3.15
